# Band transition and topological interface modes in 1D elastic phononic crystals

**DOI:** 10.1038/s41598-018-24952-5

**Published:** 2018-05-01

**Authors:** Jianfei Yin, Massimo Ruzzene, Jihong Wen, Dianlong Yu, Li Cai, Linfeng Yue

**Affiliations:** 1 Laboratory of Science and Technology on Integrated Logistics Support, National University of Defense Technology, Changsha, Hunan 410073 P.R. China; 2 School of Aerospace Engineering, Georgia Institute of Technology, Atlanta, GA 30332 USA; 3 School of Mechanical Engineering, Georgia Institute of Technology, Atlanta, GA 30332 USA

## Abstract

In this report, we design a one-dimensional elastic phononic crystal (PC) comprised of an Aluminum beam with periodically arranged cross-sections to study the inversion of bulk bands due to the change of topological phases. As the geometric parameters of the unit cell varies, the second bulk band closes and reopens forming a topological transition point. This phenomenon is confirmed for both longitudinal waves and bending waves. By constructing a structural system formed by two PCs with different topological phases, for the first time, we experimentally demonstrate the existence of interface mode within the bulk band gap as a result of topological transition for both longitudinal and bending modes in elastic systems, although for bending modes, additional conditions have to be met in order to have the interface mode due to the dispersive nature of the bending waves in uniform media compared to the longitudinal waves.

## Introduction

Phononic crystals (PCs) and acoustic metamaterials are intensively studied as means to manipulate sound or elastic waves^1–13^. Within this field, recent attention has been devoted to acoustic analogues of topological concepts in condensed matter physics such as quantum spin Hall effect^14–17^ and topological phases^18–22^. Properties of particular interest include the existence of topologically protected edge states^23–28^, which could be beneficial for a variety of applications including acoustic focusing, energy harvesting and vibration/noise control^29–33^.

The topological properties of the band structure of a material can be described in terms of topological invariants such as the Berry phase^34,35^, or the Zak phase for one-dimensional media^36,37^. Recent studies show that edge modes within the band gap exist at the interface between two PCs with different topological properties^28,31,38^. This phenomenon has been confirmed for several one-dimensional (1D) PC systems such as discrete spring-mass lattices^39^, acoustic systems^40^ and surface-water-wave system^41^. The extension of these investigations to elastic systems remains an open challenge due to more complicated dispersion nature of waves propagating in elastic structures. While previous work mainly focuses on longitudinal wave motion, this report describes theoretical studies and experimental demonstration of the existence of interface modes induced by topological transitions for both longitudinal and bending waves in 1D elastic wave guides.

## Model

**Figure 1:**
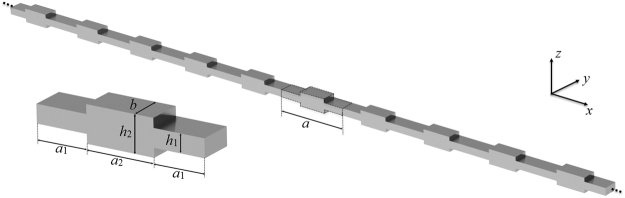
3D view of the PC and its unit cell.

Inspired by previous works on acoustic systems^40–42^, we consider a simple 1D elastic phononic crystal (PC) corresponding to a beam structure with periodically varying, step-wise cross-section as shown Fig. 1. The unit cell consists of two thinner beam components of equal length *a*_1_ sandwiching a thicker beam of length *a*_2_, thus the lattice constant of the PC is *a* = 2*a*_1_ + *a*_2_. In this work, *a* is set to be 0.15 m. The cross-section of the beam is rectangular and the material and geometrical parameters are listed in Table 1. For a structural PC, it can support propagation of both longitudinal waves and bending waves.

**Table 1:** Material and geometric properties of the PC.

*b* (mm)	*h*_1_ (mm)	*h*_2_ (mm)	Young’s modulus *E* (Pa)	Density *ρ* (kg/m^3^)	Poisson’s ratio
10	20	10	70 × 10^9^	2700	0.33

For longitudinal waves in uniform media, the wave vector *k* follows the dispersion relation *ω*^2^ = *k*^2^*E/ρ* thus it is non-dispersive. In contrast, for bending waves in beam structures, the Timoshenko model yields the dispersive relation expressed as (See Methods), which in contrast to the linear dispersion relation of longitudinal waves, is non-linear for dispersion.

1 $${\omega }^{4}+(\frac{G{k}_{t}+E}{4\rho }{k}^{2}-\frac{SG{k}_{t}}{\rho I}){\omega }^{2}+\frac{4{\rho }^{2}}{EG{k}_{t}}{k}^{4}=0$$

Normally in elastic structures, longitudinal and bending waves co-exist and couple with each other at structural junctions. In order to study the two wave types separately, a symmetric arrangement of the PC with respect to the neutral *x-y* plane is used to ensure the decoupling of longitudinal and transverse (bending) waves^43,44^. The transfer matrix (TM) method in conjunction with the Bloch theorem for periodic structures is used to calculate the dispersion relations of the PC (See Methods). Physical experiments along with numerical simulations with finite element method (FEM) are used to study the forced response of finite PC systems and demonstrate the existence of interface modes.

## Results

We start by considering the band structure of longitudinal waves for three PCs configurations defined by a geometric parameter *δ* = (2*a*_1_ − *a*_2_)/*a*, namely LC1: *δ* = −1/3 (*a*_1_ = 0.025 m), LC2: *δ* = 0 (*a*_1_ = 0.0375 m) and LC3: *δ* = 1/3 (*a*_1_ = 0.05 m) as shown in Fig. 1(a–c). It is observed that the band structure for the first three modes of LC1 and LC3 appear identical and feature two band gaps. Here we focus on the second band gap bounded by the second and third eigenmodes. As for LC2, the second and third eigenmodes become degenerate at *k* = 0 which closes the second band gap.

From the perspective of wave propagation, the band gaps are induced by wave scattering due to impedance mismatch at the structural junctions of the PC. In this report, a topological concept is used to study the band behavior of the PC. The topological property of a Bloch band can be defined by certain invariants, which, for 1D systems, can be expressed in terms of Zak phase^37^. Non-trivial Zak phase indicates the existence of edge states^45^, which was initially discussed in quantum theory^37^ and then found in condensed matter physics described by the Su-Schrieffer-Heeger (SSH) model^46^ and in acoustic systems^40^. The Zak phase quantifies the change in polarization for wave vectors across the Brillouin zone, identified after fixing an appropriate gauge. The Zak phase is numerically calculated using COMSOL for a 1D finite element model using beam element (see Methods), following the procedure described by Xiao *et al*.^40,47^. For a unit cell with symmetric geometry with respect to the central cross-section plane, they can acquire a Zak phase of either 0 or *π* depending on the value of *δ* of the PC. The Zak phase is closely related to the wave propagation in the PC by indicating the advance (Zak phase being *π*) or delay (Zak phase being 0) of the phase of the reflected wave.

**Figure 2:**
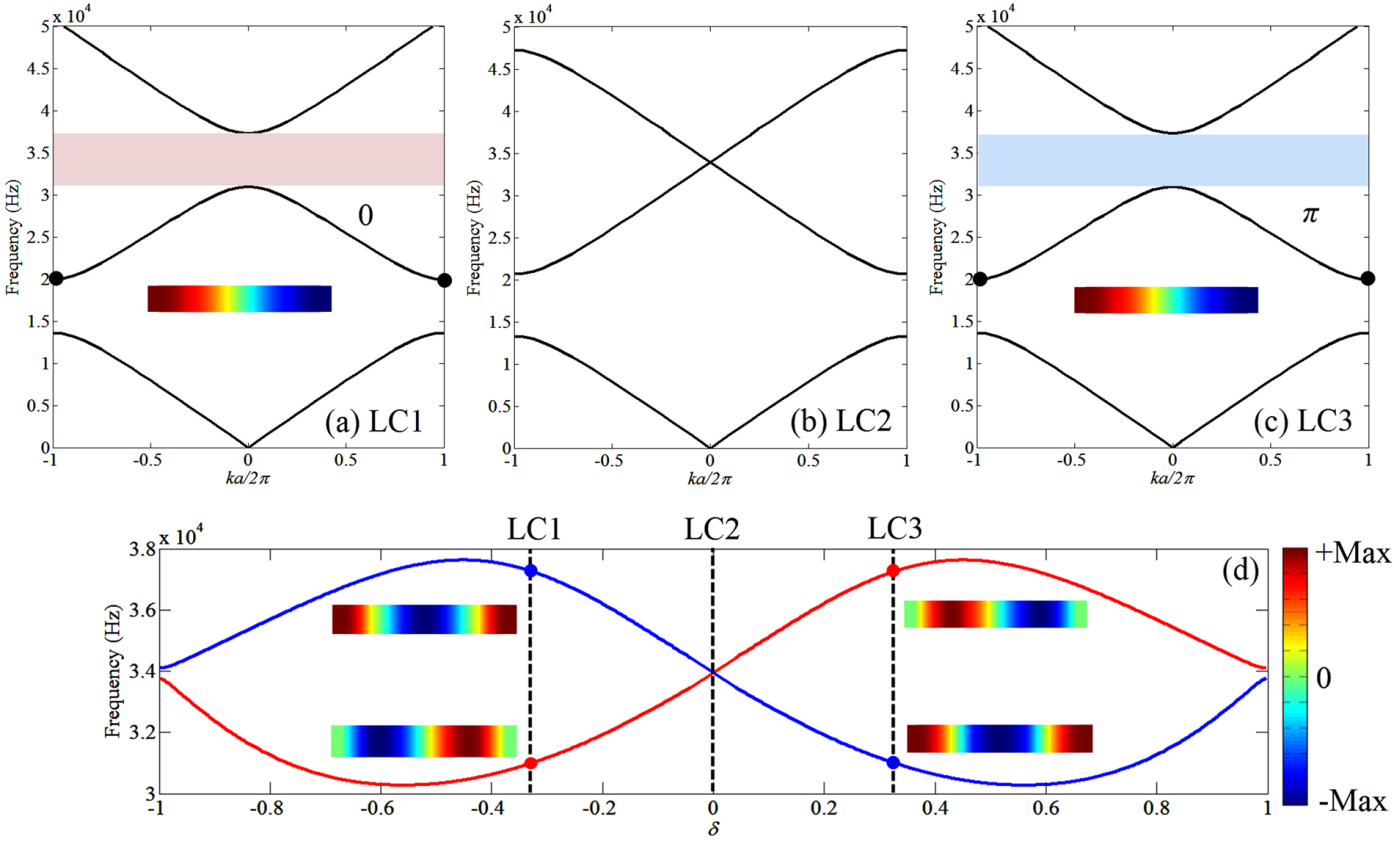
Band structure of the PC with different geometric configurations and topological transition for longitudinal waves. (**a–c**) Band structure of the longitudinal modes for LC1, LC2 and LC3. The Zak phase of the second band for LC1 is 0 and LC3 is *π*. The second band gap closes at LC2 at the center of the Brillouin zone as the two modes become degenerate. (**d**) Bounding frequencies of the second band gap at the center of the Brillouin zone with variation of the geometric parameter *δ*. The blue curve corresponds to symmetric mode with the respect of the center cross-section of the unit cell and the red curve corresponds to unsymmetric mode. The mode shapes of two bounding modes for LC1 and LC3 are shown where an inversion of mode polarization is observed at *δ* = 0. (**a,c**) the mode shape of the second eigenfrequency at the edge of the Brillouin zone for LC1 and LC3 are shown in the insets to demonstrate that when the mode shapes at the center and edge of the Brillouin zone have the same symmetry to the central cross-section plane as LC1 (bothe unsymmetric), the Zak phase is 0, otherwise is *π* as LC3.

It is marked in Fig. 2(a,c) that the Zak phase of the second band for LC1 is 0, while for LC3 it is *π*. Therefore, LC1 and LC3 are topologically distinct despite of their apparently identical band structures. This change in Zak phase is investigated further by tracking the bounding eigenfrequencies for the second band gap at *k* = 0 by varying geometric parameter *δ*. To this end, the following expression for the dispersion relations of the PC is employed (derivation see Methods):

2 $$\cos (ka)=\,\cos (\frac{\omega a}{{c}_{{\rm{L}}}})-\frac{1}{2}(\frac{{S}_{2}}{{S}_{1}}+\frac{{S}_{1}}{{S}_{2}}-2)\sin (\frac{2\omega {a}_{1}}{{c}_{{\rm{L}}}})\sin (\frac{\omega {a}_{2}}{{c}_{{\rm{L}}}})$$

where ${C}_{{\rm{L}}}=\sqrt{E/{\rho }}$ is the longitudinal wave speed, while and *S*_1_, *S*_2_ respectively denote the cross-section areas of *a*_1_ and *a*_2_. The two bounding frequencies are associated with two wave modes whose variation in terms of parameter *δ* are shown in Fig. 2(d). The blue curve corresponds to the eigenmode that is symmetric with respect of the center cross-section plane while the red curve represents unsymmetric mode. The shape of the two modes are shown in the insets of Fig. 2(d). With the increase of $\delta$ from −1 to 1, the second band gap closes at LC2 where an accidental degeneracy occurs. Then the band gap reopens, with a switch of mode polarization: for LC1, the lower bounding mode is unsymmetric while the upper bounding mode is symmetric. The opposite phenomenon is observed for LC3. The inversion of the mode types occurs at LC2, which corresponds to the transition of Zak phase from 0 to *π* for the second band. The degeneracy condition yields that *δ* = 0 resulting in the inversion frequency follows $\omega a/{c}_{{\rm{L}}}=2\pi$ s.t. 33,945 Hz.

Figure 2(a,c) also show the mode shape of the second eigenmode at the edge of the Brillouin zone (black dots) for LC1 and LC3 in the insets and it is observed that they have the same type of mode polarization (both unsymmetric) thus no inversion occurs in contrast to the modes at the center of the Brillouin zone. By comparing the mode shapes at the center and edge of the Brillouin zone, it is found that when the second eigenmode of the same geometric configuration at center and edge of the Brillouin zone have the same type of mode polarization, the Zak phase of this band is 0, otherwise it is *π*. This conclusion is also confirmed in ref.^40^.

**Figure 3:**
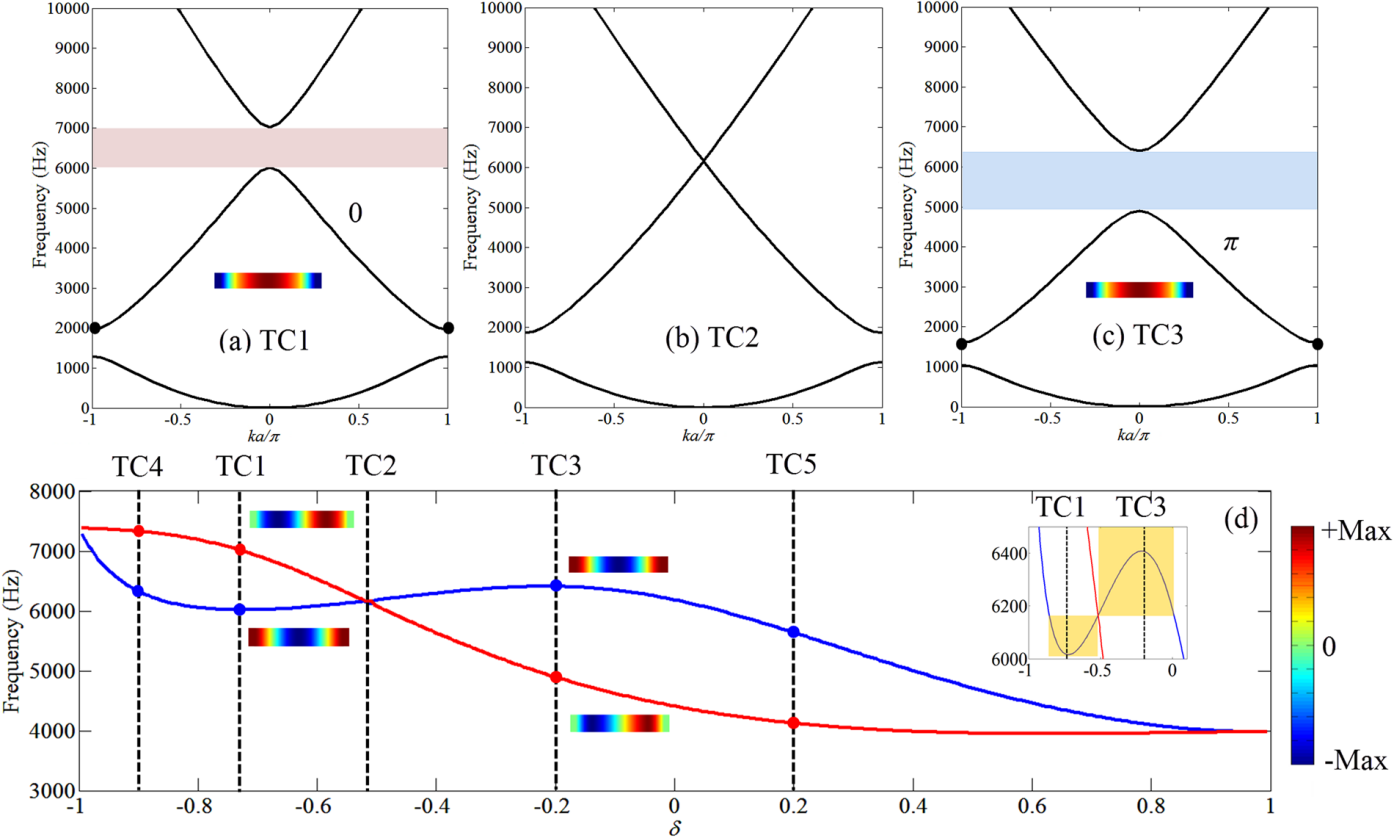
Band structure of the PC with different geometric configurations and topological transition for bending waves. (**a**–**c**) Band structure of the bending modes for TC1-TC3. The Zak phase of the second band for TC1 is 0, TC3 is *π*. The second band gap closes at TC2 at the center of the Brillouin zone as the two modes become degenerate. (**d**) Bounding frequencies of the second band gap at the center of the Brillouin zone with variation of the geometric parameter *δ*. The blue curve corresponds to symmetric mode with the respect of the center cross-section of the unit cell and the red curve corresponds to unsymmetric mode. The mode shapes of two bounding modes for TC1(TC4) and TC3(TC5) are shown where an inversion of modes can be observed at TC2(*δ* = −0.5173). The yellow regions in the enlarged figure indicate the condition on geometric range for constructing interface mode with two PCs with different Zak phases. (**a**,**c**) The mode shape of the second eigenfrequency at the edge of the Brillouin zone for TC1 and TC3 are shown in the insets to demonstrate that when the mode shapes at the center and edge of the Brillouin zone have the same symmetry to the center cross-section plane as TC1, the Zak phase is 0, otherwise is *π* as TC3.

For bending waves, the topological transition point also exists as seen in Fig. 3(d), where the bounding frequencies of the second band gap are again shown as function of the geometric parameter *δ*. Four configurations of the PC are chosen for analysis, namely TC1: *δ* = −11/15 (*a*_1_ = 0.01 m), TC2: *δ* = −0.5173 (*a*_1_ = 0.0181 m), TC3: *δ* = −0.2 (*a*_1_ = 0.03 m), TC4: *δ* = −0.8933 (*a*_1_ = 0.004 m) and TC5: *δ* = 0.2 (*a*_1_ = 0.045 m). The band structures of TC1-TC3 along with their Zak phase of the second band are shown in Fig. 3(a–c) where the Zak phase for TC1 is 0 and for TC3 is *π*. With the increase of *δ*, the second band closes at TC2 and reopens with the Zak phase changing from 0 to *π*.

In contrast to the longitudinal results in Fig. 2(d), it is observed that for the unsymmetric mode (red line in Fig. 3(d)), the eigenfrequency monotone decreases with the increase of *δ* while the symmetric mode (blue line) doesn’t have such monotone behavior. As a result, one can still observe the band closing and reopening at the transition point, which however does not occur at *δ* = 0 as the longitudinal model. This behavior is related to the fact that the dispersion relations of bending waves in uniform beam is non-linear as described by eq. (1).

**Figure 4:**
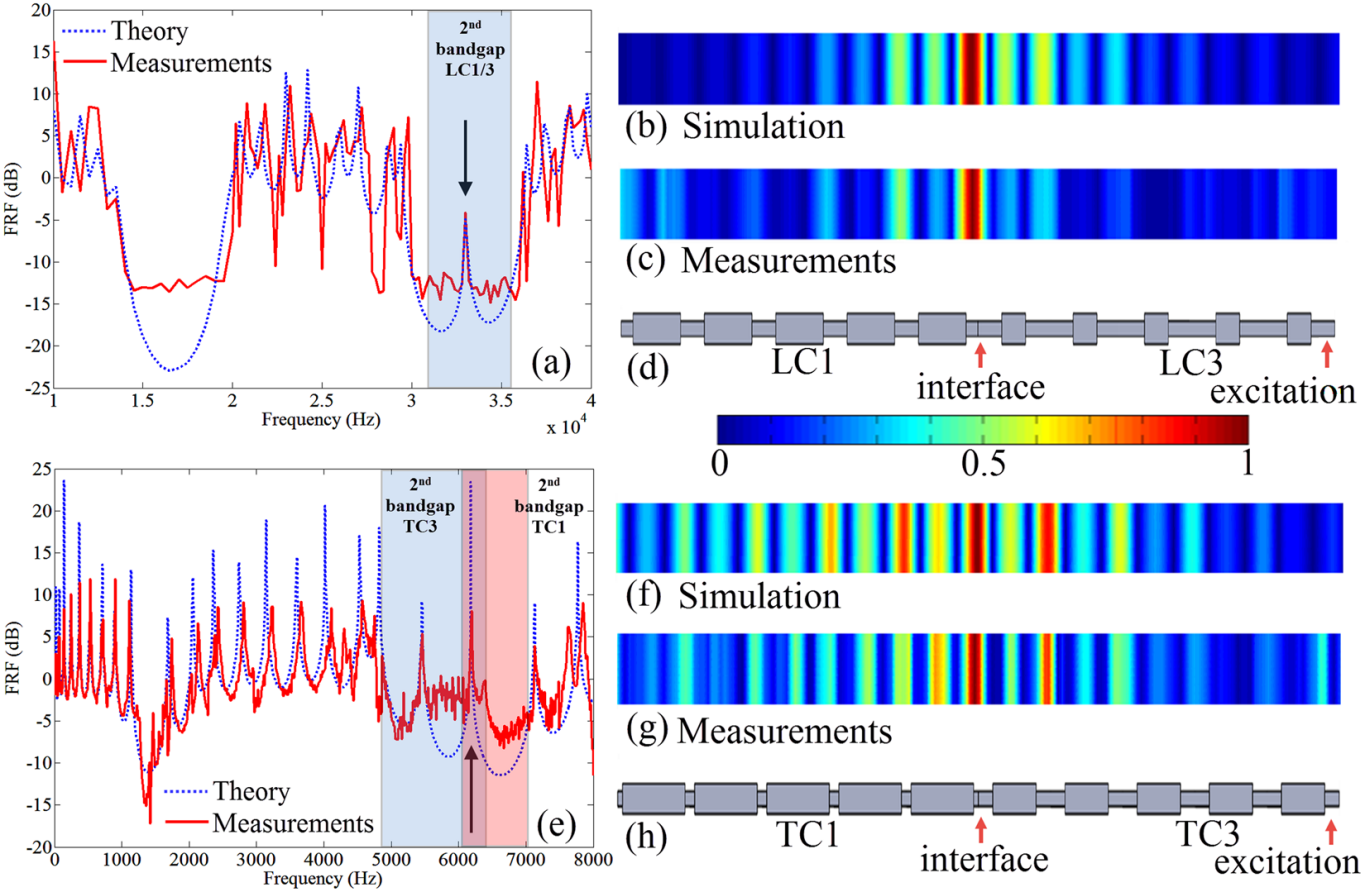
Forced response of the finite system comprised of two PCs with different topological properties obtained from measurements in comparison with numerical simulations using FEM. (**a**) FRF of (**d**) LC1 + LC3 where the beam is excited at the right end and velocity response picked up at the other end (the dashed blue lines represent simulated results using FEM and the solid red lines are measured results). A transmission peak at 33,100 Hz is observed within band gap region (colored area) indicating the existence of the interface mode. (**b**,**c**) the longitudinal normalized velocity field obtained from simulations (**b**) and measurements (**c**) at the peak frequency, respectively. (**e**) FRF of (**h**) TC1 + TC3 where the beam is excited at the right end and velocity response picked up at the other end (the dashed blue lines represent simulated results using FEM and the solid red lines are measured results). Transmission peak at 6,162 Hz occurs within the common band gap region (overlapped colored area). (**f**,**g**) The bending normalized velocity field at the peak frequency obtained from simulation(**f**) and measurements(**g**) demonstrating the interface mode.

Next, we construct a finite system by connecting two PCs with different topological properties, with the objective of demonstrating the occurrence of an interface mode resulting from the topological transition. For the longitudinal wave case, a system comprising 5 cells of LC1 connecting 5 cells of LC3 is used as shown in Fig. 4(d). The Frequency Response Function (FRF) of the system is obtained for both experimental measurements and numerical simulations (See Methods). In the experiments, the system is excited at one end (right) of the PC using a PZT patch with single frequency sinusoidal signal and sweeping over the frequency range of interest for each measurement and the response in terms of velocity is picked up at the other end of the PC(left). As shown in Fig. 4(a), a transmission peak at 33,100 Hz (0.4% error compared with theory) in the FRF is clearly observed within the second band gap for the two PCs. The mode associated with this peak is localized at the interface. In order to further confirm this, the corresponding normalized velocity field along the finite beam is plotted for both simulation and measured data as in Fig. 4(b,c), respectively. It is shown that the longitudinal motion is concentrated at the interface and decays rapidly away from it in both directions.

**Figure 5:**
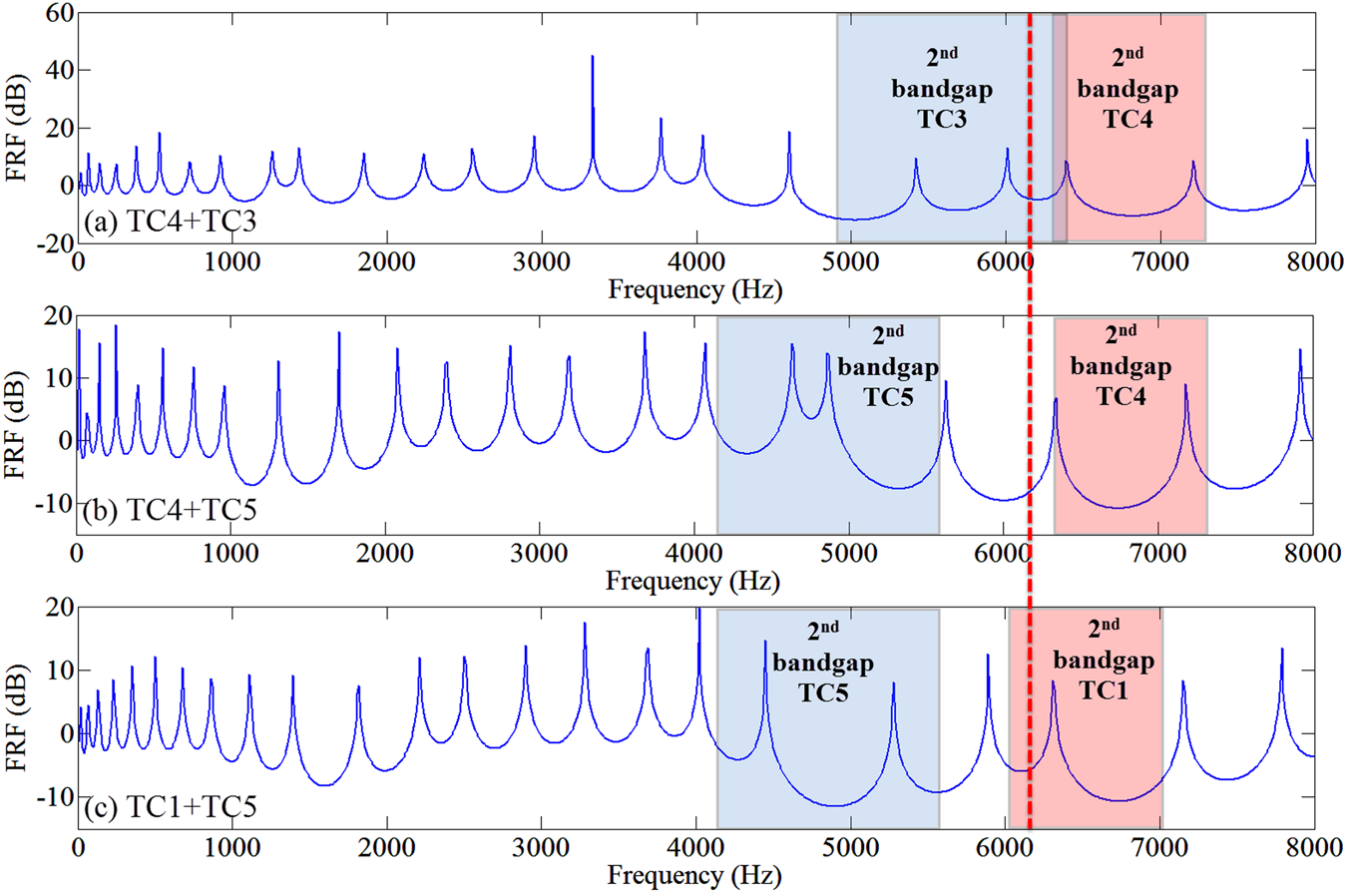
FRFs for finite PC systems for which the bending interface mode cannot be observed. (**a**) TC3 + TC4: two PCs have overlapping band gap region but the topological transition frequency (marked by the red dash line) is not in the overlapping band gap region. (**b**,**c**) TC5 + TC4 and TC5 + TC1: two PCs don’t have overlapping band gap. For all cases, the two PCs are topological distinct (red band gap region corresponds 0 Zak phase for the lower bulk band and blue region *π*). The transmission peak induced by interface mode cannot be observed in all cases.

For bending waves, a system with 5 cells of TC1 connecting 5 cells of TC3 is constructed as shown in Fig. 4(h). In contrast with the longitudinal test, the PC is now excited using a shaker by a white noise. The transmission peak within the common band gap region is observed in Fig. 4(e) and the displacement field plots in Fig. 4(f,g) again confirm the existence of the interface mode. Since the frequency of the interface mode corresponds to the mode transition frequency (6,162 Hz for bending model), to observe the interface mode, the two PCs with different topological properties must ensure that the mode transition frequency is within the frequency range of both their second band gap as the model with TC1 + TC3. As for longitudinal model, this requirement is always satisfied as long as the two PCs have different topological properties. Based on this, the inset of Fig. 3(d) marks out the region for the interface mode to be created by the hybridization of two PCs. The examples shown in Fig. 5 illustrate numerically that without meeting the conditions that 1) two PCs are topologically distinct; 2) the topological transition frequency falls within the overlap band gap of the two PCs, the interface mode cannot be achieved.

**Figure 6:**
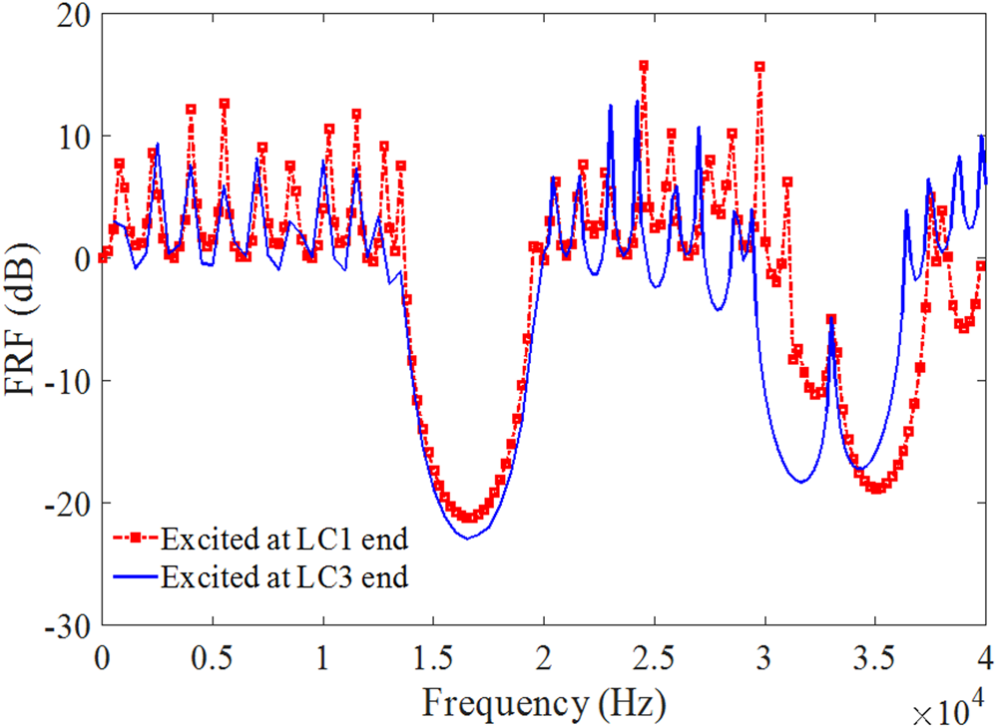
FRFs for LC1 + LC3 systems where excitation is applied at LC1 and LC3 end respectively and the response is picked up at the other end. The interface mode at 33.1 kHz is observed in both cases.

It is to be noted that the interface mode observed in Fig. 4(a) differs from that have been previously studied as ‘boundary mode’^48,49^ which is attributed to boundary conditions, the coupling of local resonances or the position of excitation, therefore, such interface mode is robust against geometric boundary conditions or the direction of wave transmission. To demonstrate the robustness of the interface state, Fig. 6 shows the FRFs for the LC1 + LC3 model which is excited at the two ends respectively and in both cases, the interface mode is observed at the same frequency represented by the transmission peak despite of the difference in FRF at some other frequencies.

**Figure 7:**
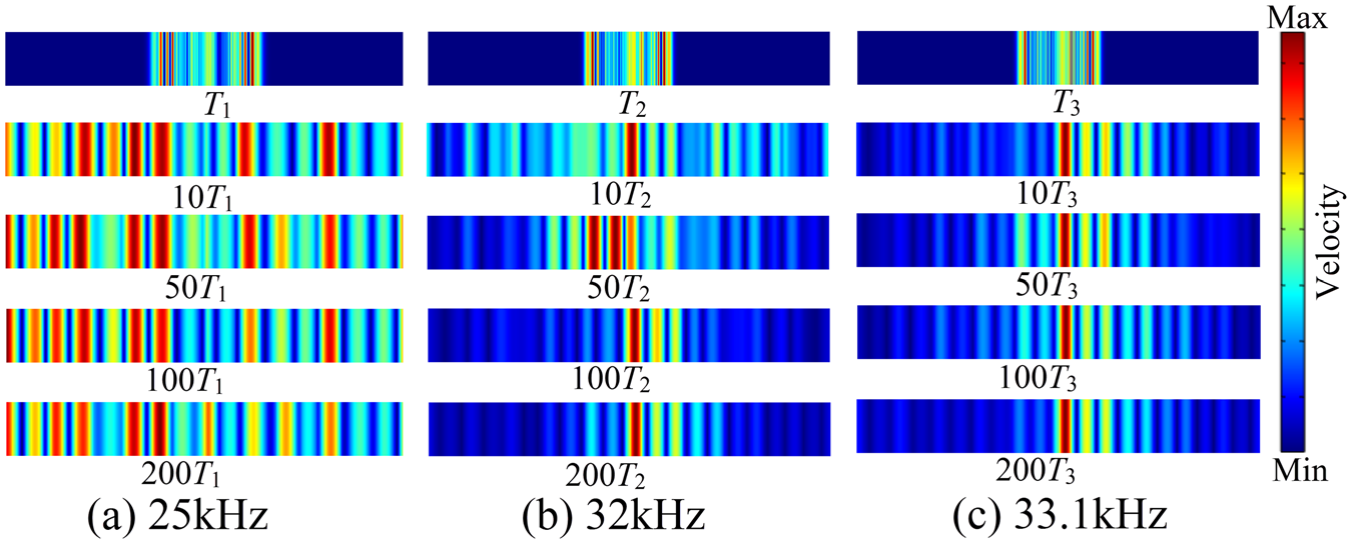
Forced response of LC1 + LC3 system in time domain at different time instances where the frequency of excitation is at (**a**) 25 kHz, (**b**) 32 kHz and (**c**) 33.1 kHz.

In addition, we consider the same longitudinal model in time domain to demonstration the effect of the interface mode on wave propagation. A harmonic prescribed in-plane displacement with an amplitude of 10^–4^ m is applied at the geometric interface of LC1 + LC3 model. Three frequencies are chosen for analysis at 25 kHz (in the second band), 32 Hz (in the second band gap) and 33.1 kHz (at the interface mode frequency in the second band gap). The velocity fields at different time instances are plotted as shown in Fig. 7(a–c). It is shown that when the excitation frequency is not in the band gap, the wave can propagate along the beam thus response peaks are spread across the beam as in Fig. 7(a) while when the excitation is within the band gap, the wave cannot propagate in the beam freely thus the response peak is mainly concentrated at the excitation position as in Fig. 7(b) and (c). Compared with normal band gap frequency, when the excitation is at the interface mode frequency, the response seems to be more concentrated with less energy leakage although the difference can be hardly seen when the time is long enough where the response reaches steady state.

## Conclusions

In summary, this report illustrates both theoretically and experimentally the topological transition for a 1D elastic periodic beam as a waveguide. We have evaluated the topological properties of the 1D system for both longitudinal and bending waves which are quantified by the Zak phase estimation. A band inversion for variations of a relevant geometric property is observed as defined by a corresponding change in Zak phase. Based on this finding, we have further demonstrated the existence of interface mode within the bulk band gap by constructing a finite system with two PCs of different topological phases for both longitudinal and bending waves. It is also noted that the dispersive nature of the bending wave in uniform media leads to different band inversion characteristics compared to the longitudinal waves. Specifically, the numerical examples in this report show that for bending waves the interface mode exists only when the band inversion frequency is within the overlapping band gap region for the two PCs connected in series. The interface mode is robust against excitation and exhibited higher energy concentration at the interface compared to normal bandgap frequency when excitation is applied at the interface.

This report extends the study of topological concept to elastic systems that are characterized by both dispersive and non-dispersive wave properties. The analysis and results presented herein may be applied to other 1D or 2D periodic structural systems whereby topological interfaces are strategically placed to achieve wave localization and isolation, with potential application to vibration control and energy harvesting.

## Methods

### Theories

We have implemented the transfer matrix method for the analysis of dispersion for longitudinal and bending modes. The wave fields for the *n*^th^ unit cell in terms of displacement can be written as ${u}_{nm}(x,t)$ for axial direction and ${w}_{nm}(x,t)$ for lateral direction where *m* = 1, 2, 3 representing the three components of the unit cell. The propagation of longitudinal waves follows eq. (3):

3 $$\frac{\rho }{S}\frac{{\partial }^{2}u}{\partial {t}^{2}}=\frac{\partial }{\partial x}[E\frac{\partial u}{\partial x}]$$

where *ρ*, *E* and *S* are the density, Young’s modulus and cross-section area of the beam, while for bending waves, the Timoshenko beam model is employed leading to the following characteristic equation^48^

4 $$\frac{EI}{\rho S}\frac{{{\rm{\partial }}}^{4}w(x,t)}{{\rm{\partial }}{x}^{4}}-\frac{I}{S}(1+\frac{E}{{k}_{t}G})\frac{{{\rm{\partial }}}^{4}w(x,t)}{{\rm{\partial }}{x}^{2}{\rm{\partial }}{t}^{2}}+\frac{{{\rm{\partial }}}^{2}w(x,t)}{{\rm{\partial }}{t}^{2}}+\frac{\rho I}{{k}_{t}GS}\frac{{{\rm{\partial }}}^{2}w(x,t)}{{\rm{\partial }}{t}^{2}}=0$$

In eq. (4), *I*, *G* and *k*_*t*_ = 1/1.2 denote second moment of area, shear modulus and shear correction for a rectangular cross section.

Considering the *n*^th^ unit cell, at the boundaries and junctions within the unit cell at *x* = *na* + *a*_1_, *x* = *na* + *a*_1_ + *a*_2_ and *x* = (*n* − 1)*a*, we apply the continuity conditions to obtain the field transfer matrix between *n*^th^ and (*n* − 1)^th^ unit cell:

5 $${{\boldsymbol{\Psi }}}_{n}={\bf{T}}{{\boldsymbol{\Psi }}}_{(n-1)}$$

Due to the periodicity of the infinite structure in the *x* direction, the vector ${{\boldsymbol{\Psi }}}_{n}$ must satisfy the Bloch theorem

6 $${{\boldsymbol{\Psi }}}_{n}={e}^{ika}{{\boldsymbol{\Psi }}}_{(n-1)}$$

where *k* is the Bloch wave vector written in scalar form for the 1D system. It follows that the eigenvalues of the infinite periodic structures are the roots of the determinant:

7 $$|{\bf{T}}-{e}^{ika}{\bf{I}}|=0$$

Eq. (7) is a standard eigenvalue problem from which the dispersion relations between *k* and *ω* can be obtained.

For longitudinal wave, a closed-form of dispersion relations can be further derived by taking ${u}_{nm}(x,t)={A}_{1}{e}^{i\omega x/{c}_{{\rm{L}}}}+$ ${A}_{2}{e}^{-i\omega x/{c}_{{\rm{L}}}}$ into eq. (3) at corresponding boundary/ junctions and by calculating the determinant of $({\bf{T}}-{e}^{ika}{\bf{I}})$ it gives:

8 $$\cos (ka)=\,\cos (\frac{2\omega {a}_{1}}{{c}_{{\rm{L}}}})\cos (\frac{\omega {a}_{2}}{{c}_{{\rm{L}}}})-\frac{1}{2}(\frac{{S}_{2}}{{S}_{1}}+\frac{{S}_{1}}{{S}_{2}})\sin (\frac{2\omega {a}_{1}}{{c}_{{\rm{L}}}})\sin (\frac{\omega {a}_{2}}{{c}_{{\rm{L}}}})$$

Since *a* = 2*a*_1_ + *a*_2_, eq. (8) can be simplified to eq. (2).

We use finite element method in conjunction with transfer matrix method to calculate the forced response of the finite PC system. We consider each unit cell to be governed by the following dynamic stiffness formulation:

9 $${\bf{Dq}}={\bf{f}}$$

where ${\bf{D}}={\bf{K}}-{\omega }^{2}{\bf{M}}$ is the dynamic stiffness matrix, **q** and **f** are the vectors of nodal dofs and forces, **K** and **M** are the stiffness and mass matrices.

Eq. (9) can be rearranged in matrix form as:

10 $$[\begin{array}{cc}{{\bf{D}}}_{{\rm{LL}}} & {{\bf{D}}}_{{\rm{LR}}}\\ {{\bf{D}}}_{{\rm{RL}}} & {{\bf{D}}}_{{\rm{RR}}}\end{array}][\begin{array}{c}{{\bf{q}}}_{{\rm{L}}}\\ {{\bf{q}}}_{{\rm{R}}}\end{array}]=[\begin{array}{c}{{\bf{f}}}_{{\rm{L}}}\\ {{\bf{f}}}_{{\rm{R}}}\end{array}]$$

where the subscript L and R represent the left and right hand sides of the element. For uniform waveguides, we have

11 $${{\bf{D}}}_{{\rm{LL}}}^{{\rm{T}}}={{\bf{D}}}_{{\rm{LL}}},\,{{\bf{D}}}_{{\rm{RR}}}^{{\rm{T}}}={{\bf{D}}}_{{\rm{RR}}},{{\bf{D}}}_{{\rm{LR}}}^{{\rm{T}}}={{\bf{D}}}_{{\rm{RL}}}$$

where (*)^T^ is the transpose.

Using the transfer matrix to describe the continuity of displacement and force equilibrium of adjacent segments gives

12 $$[\begin{array}{c}{{\bf{q}}}_{{\rm{L}}}^{(n+1)}\\ {{\bf{f}}}_{{\rm{L}}}^{(n+1)}\end{array}]=[\begin{array}{c}{{\bf{q}}}_{{\rm{R}}}^{(n)}\\ {{\bf{f}}}_{{\rm{R}}}^{(n)}\end{array}]={\bf{T}}[\begin{array}{c}{{\bf{q}}}_{{\rm{L}}}^{(n)}\\ {{\bf{f}}}_{{\rm{L}}}^{(n)}\end{array}]$$

The transfer matrix **T** can be thus found in the terms of the elements of the dynamic stiffness matrix by:

13 $${\bf{T}}=[\begin{array}{cc}-{{\bf{D}}}_{{\rm{LR}}}^{-1}{{\bf{D}}}_{{\rm{LL}}} & {{\bf{D}}}_{{\rm{LR}}}^{-1}\\ -{{\bf{D}}}_{{\rm{RL}}}+{{\bf{D}}}_{{\rm{RR}}}{{\bf{D}}}_{{\rm{LR}}}^{-1}{{\bf{D}}}_{{\rm{LL}}} & -{{\bf{D}}}_{{\rm{RR}}}{{\bf{D}}}_{{\rm{LR}}}^{-1}\end{array}]$$

Inversely, the dynamic stiffness matrix **D** can be calculated given the transfer matrix **T**.

14 $${\bf{D}}=[\begin{array}{cc}-{{\bf{T}}}_{{\rm{LR}}}^{-1}{{\bf{T}}}_{{\rm{LL}}} & {{\bf{T}}}_{{\rm{LR}}}^{-1}\\ -{{\bf{T}}}_{{\rm{RL}}}+{{\bf{T}}}_{{\rm{RR}}}{{\bf{T}}}_{{\rm{LR}}}^{-1}{{\bf{T}}}_{{\rm{LL}}} & -{{\bf{T}}}_{{\rm{RR}}}{{\bf{T}}}_{{\rm{LR}}}^{-1}\end{array}]$$

For a finite system with *N* unit cells in a chain, the global dynamic stiffness can be obtained from assembling the elementary stiffness from cells 1 to *N*.

### Numerical calculation of the Zak phase

This work follows the method proposed by by Xiao *et al*.^40,45^ to calculate the Zak phase for bulk bands of the PC. For the *n*^th^ Bloch band of a 1D PC, its Zak phase ${{\theta }}_{{\rm{n}}}^{{\rm{Zak}}}$ is given by:

15 $${{\theta }}_{n}^{{\rm{Zak}}}={\int }_{-\pi /a}^{\pi /a}[i{\int }_{unitcell}\frac{1}{2\rho {c}^{2}}dx{\xi }_{n,k}^{\ast }(x)\partial {\xi }_{n,k}(x)]dk$$

where ${\xi }_{n,k}(x)$ is the cell-periodic Bloch eigenfunction for a given wavenumber *k*. For the *n*^th^ band and the longitudinal/bending wave field ${U}_{n,k}(x)/{W}_{n,k}(x)={\xi }_{n,k}(x)\exp (ikx)$. The factor $1/(2\rho {c}^{2})$ is the weight function for elastic systems.

The eigenfunction of the cell-periodic is numerically calculated using COMSOL with meshes of beam elements. The Zak phase is then calculated using a discretized form of eq. (15) after extracting the eigenfunctions from COMSOL.

16 $${{\theta }}_{n}^{{\rm{Zak}}}=-\mathrm{Im}\sum _{i=1}^{N}\mathrm{ln}[{\int }_{unitcell}\frac{1}{2\rho {c}^{2}}dx{\xi }_{n,{k}_{i}}^{\ast }(x)\partial {\xi }_{n,{k}_{i+1}}(x)]$$

### Experimental set-up

The periodic beam used for testing is machine cut out of one piece of Aluminum material to ensure geometric accuracy. The beams are suspended using bungee ropes to simulate free boundary conditions.

**Figure 8:**
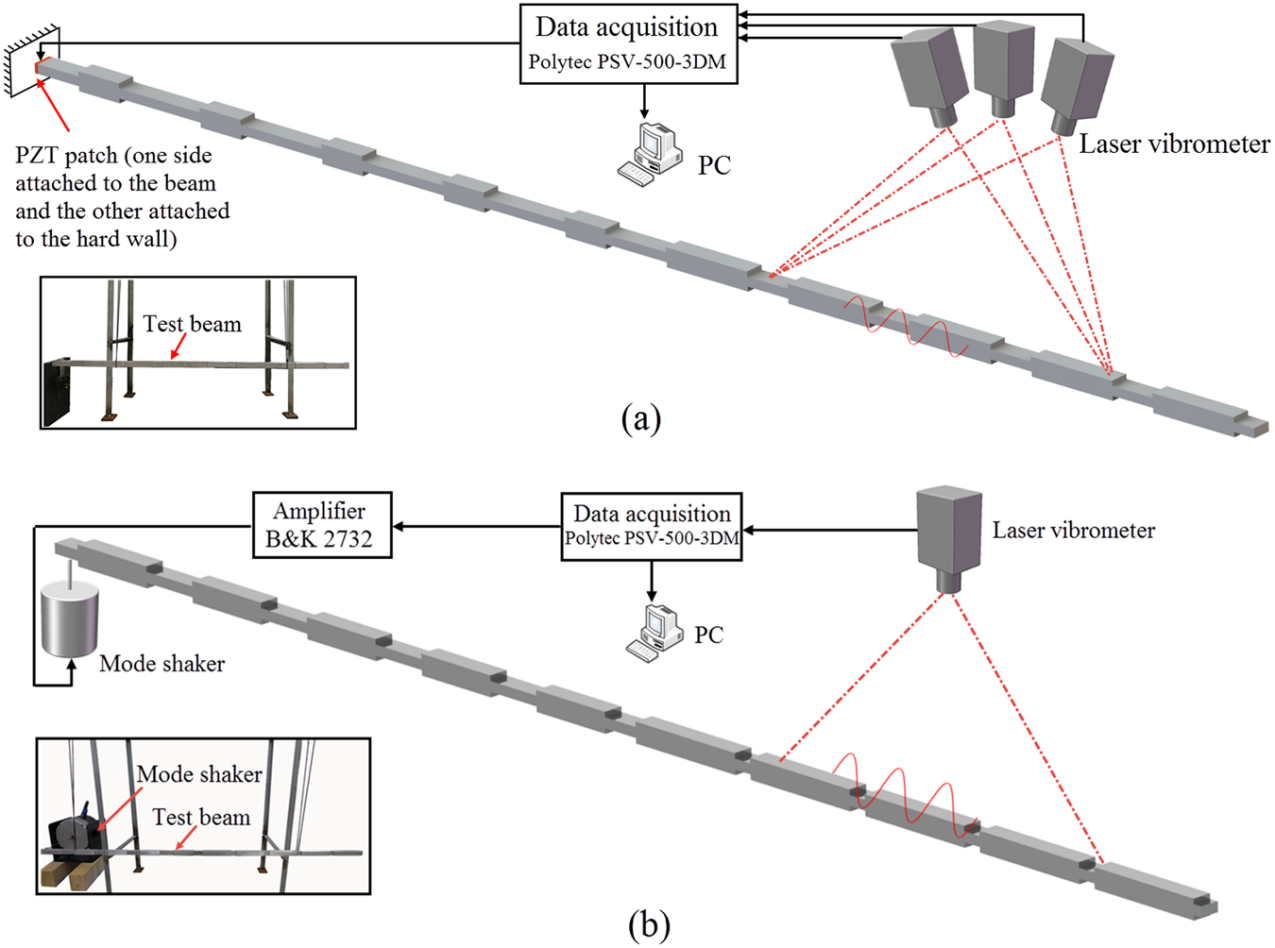
Experimental set-up schematic and photo. (**a**) Longitudinal wave testing set-up; (**b**) bending wave testing set-up. The insets show the photos of experimental set-up.

For the longitudinal wave test, the beam is excited using a PZT patch (Type: E-SOUND PZT-5; Dimensions: 20 mm × 10 mm × 2 mm) with one side attached onto one end of the beam and the other side mounted onto the wall as shown in Fig. 8(a). The other end of the beam is free. A single frequency sinusoidal signal is used in one measurement and the FRF is obtained through multiple measurements by sweeping excitation frequency over the interested frequency range. For the bending wave test, the beam is excited using a mode shaker at one end of the beam as shown in Fig. 8(b). The FRF can be obtained in single measurement using a white noise signal as excitation. The vibration response along the beam is measured using a Polytec 3D scanning laser Doppler vibrometer which provides accurate 3D dynamic motion vectors. For longitudinal wave measurement, we employ three laser heads to measure the instantaneous vibratory velocity in the axial direction, while for bending waves (Fig. 8(a)), we use only one laser head to measure the transverse motion of the beam (Fig. 8(b)). The measured results generally are in good agreement with the FEM results, and typically capture all band gaps and response peaks except for those within the band gap ranges where a higher FRF than FEM results is measured. This error is attributed to the background vibration level of the structural system.

### Data availability

The data that support the findings of this study are available from the corresponding author upon reasonable request.
